# Exercise intensities modulate ACE2/MasR/eNOS pathway in male Wistar rat's lung

**DOI:** 10.14814/phy2.15803

**Published:** 2023-09-04

**Authors:** Yani Medina Lestari, Vita Murniati Tarawan, Achadiyani Achadiyani, Putri Teesa Radhiyanti, Hamidie Ronald Daniel Ray, Ronny Lesmana, Hanna Goenawan

**Affiliations:** ^1^ Biomedical Science Master Program, Faculty of Medicine Universitas Padjadjaran Bandung Indonesia; ^2^ Department of Biomedical Science, Faculty of Medicine Universitas Padjadjaran Jatinangor Indonesia; ^3^ Faculty of Sport and Health Education Universitas Pendidikan Indonesia Bandung Indonesia; ^4^ Central Laboratory Universitas Padjadjaran Jatinangor Indonesia

**Keywords:** ACE2, eNOS, exercise, MasR

## Abstract

Specific exercise intensities could improve lung vascular function by increasing nitric oxide (NO). The ACE2/MasR/eNOS axis is one of the pathways facilitating NO synthesis. This study examines the effect of different intensities of aerobic training on the ACE2/MasR/eNOS axis and histology of lung muscular arteries. Male Wistar rats were used in this study and randomized into control and exercise groups receiving low‐, moderate‐, and high‐intensity training. The training was conducted for 30 min daily, five times a week, for 8 weeks. We observed that different exercise intensities affect the ACE2/MasR/eNOS pathway differently. Compared to control, high‐intensity aerobic exercise significantly increased ACE2, Mas receptor (MasR), and eNOS mRNA expressions (*p* < 0.01). Moderate‐intensity exercise significantly increased MasR and eNOS mRNA expressions compared to the control (*p* < 0.05), and this intensity also increased ACE2 mRNA but not significantly. Low‐intensity exercise increased ACE2, MasR, and eNOS mRNA expressions but not significantly. Low‐, moderate‐, or high‐intensity exercises reduced the medial wall thickness of the lung muscular arteries but not significantly. In conclusion, high‐intensity exercise may induce NO synthesis in the lung by increasing mRNA expression of ACE2, MasR, and eNOS without decreasing the medial wall thickness of the muscular artery. Thus, high‐intensity exercise may be the optimal intensity to improve NO synthesis and vascular function in the lung.

## 1 INTRODUCTION

The lung is a vital organ in the respiratory system facilitating oxygen and carbon dioxide exchange. As an organ, the lung's function must be optimized to meet oxygen needs. Mechanisms to maximize lung function will contribute to an increase in VO_2_ max, indicating an individual's physical fitness (Buttar et al., 2019; Ranković et al., 2010). Vasodilators, such as nitric oxide (NO), could increase lung perfusion through vasodilation. Improved lung perfusion can contribute to increased VO_2_ max.

Angiotensin‐converting enzyme 2 (ACE2), Mas receptor (MasR), and endothelial nitric oxide synthase (eNOS) or ACE2/MasR/eNOS pathway mediate the vasodilatory effect of NO (Rabelo et al., 2011; Savoia et al., 2020). There are several functions of NO in the lungs, for example, improving ventilation‐perfusion mismatch by increasing lung perfusion and inducing maturation of Type I and II alveolar cells during the perinatal period (Coulombe et al., 2019; Signori et al., 2022). NO also has an anti‐inflammatory effect and could inhibit the proliferation of vascular smooth muscle cells (VSMC) (Tanner et al., 2000; Wallace, 2005). NO inhibits the proliferation of VSMC, hence neointimal hyperplasia. Several mechanisms are involved in the inhibition process; one is NO, increasing the ubiquitination and decreasing ubiquitin‐conjugating enzyme (UbcH10) levels in VSMC. UbcH10 plays a role in the metaphase‐to‐anaphase transition (Tsihlis et al., 2011). NO also inhibits the cell cycle, mainly at the G1/S transition phase (Al‐omran et al., 2013).

Physical activities, such as exercise, improve fitness and increase NO availability (Oral, 2021). Exercise is categorized into low‐, moderate‐, and high‐intensity (Pescatello et al., 2009). Exercise could increase AMPK by changing the ratio of AMP/ATP and ADP/ATP (Pirkmajer et al., 2021). Later, AMPK contributes to phosphorylate ACE2 (Guignabert et al., 2018; Papadopoulos et al., 2022; Zhang et al., 2018). ACE2 will convert Angiotensin II (Ang‐II) into Angiotensin‐(1–7) [Ang‐(1–7)]. Furthermore, Ang‐(1–7) will activate the Mas receptor and phosphorylate eNOS through the PI3K/Akt signaling pathway (McKinney et al., 2014; Sampaio et al., 2007; Silva et al., 2020). Then, eNOS will catalyze the conversion of L‐arginine (L‐Arg) to L‐citrulline (L‐Cit) and NO. As a vasodilator, NO will diffuse into VSMC and activate sGC, then convert GTP to cGMP. Increased cGMP will activate protein kinase‐G (PKG) and stimulate vasodilation (Gallardo‐Ortíz et al., 2020).

However, the effect of various exercise intensities on NO‐inducing genes and their impact on decreasing arterial wall thickness in the lung is still varied. The study aims to determine the optimal exercise intensity in generating NO and reducing the arterial wall thickness in male Wistar rats. We postulate that different exercise intensities can result in different gene expression responses related to vasodilation and remodeling of lung muscular arteries. This study uses three exercise intensities to understand the effects of exercise intensities on lung function. Previous studies had only used one to two exercise intensities. The results of this study are expected to show the most beneficial intensity of aerobic exercise for pulmonary vascular function.

## 2 METHODS

### 2.1 Animals

Male Wistar rats (*Rattus norwegicus*), aged 8–12 weeks, weighed 200–225 grams, were used in this study (five rats were used in each group). Rats were given a normal chow diet and water ad libitum and placed in standard cages. This protocol was approved by the Research Ethics Committee of Padjadjaran University (1256/ UN6.KEP/EC/2022).

### 2.2 Exercise protocol

There were four groups used in this study, namely sedentary control; low‐intensity training (treadmill speed of 10 m/min); moderate‐intensity training (treadmill speed of 20 m/min); and high‐intensity training (treadmill speed of 30 m/min). Twenty male rats were randomized, acclimatized to the laboratory environment for 2 weeks, and then habituated to treadmill running for 2 weeks. A modified rat treadmill machine (IDEAS) was used in this study. Eight weeks of treadmill training were performed (5 days a week, 30 min per day). Exercise intensities were based on the accumulation of lactate levels, as studied in the previous study (Lesmana et al., 2016). Rats were sacrificed on the last day of exercise by inhalation of isoflurane 5% or more until 1 min after breathing stopped (Leary et al., 2020). We used isoflurane inhalation based on the ethical approval as issued. Liquid nitrogen was used to freeze the lung tissue sample. Then, the sample was stored at −80°C for semiquantitative PCR examination. The other part of the lung tissue was held in a formalin solution for histological analysis.

### 2.3 Histology

Left lung tissue was fixed in 10% formalin solution and embedded in paraffin. Five micrometer tissues was stained using hematoxylin–eosin. ZEISS Axio Imager Z.2 microscope (Carl Zeiss Microscopy GmbH) with 400× magnification was used to visualize the sample. Images were captured using Axiocam MR R3 and quantified using ImageJ software. The morphometric examination was performed on the lung artery with an external diameter of 20–150 μm. The medial wall thickness of the muscular artery (%WT) was calculated using the formula: (external diameter‐internal diameter)/external diameter × 100. Randomly five arteries were selected from each subject, and the average was calculated (Yu et al., 2022).

### 2.4 mRNA extraction and semiquantitative PCR

We used the right lung tissue for PCR examination. TRIsure reagent (Bioline) was used to extract the lung tissue mRNA. Multimode Microplate Reader at 268/280 nm absorbance spectrophotometry (M200 Pro, Tecan) was used to quantify the mRNA, and One‐Step RT PCR Kit (Bioline) was used to run the semiquantitative PCR. GAPDH expression was used as the internal control to normalize the PCR bands. To visualize and quantify the PCR bands, we used BluePad Detection System and ImageJ software. Table 1 shows the primer sequences used in the PCR process.

**TABLE 1:** Primers used in PCR.

Gene	Sequences	Product size (bp)	Annealing (°C)	Cycle	References
ACE2	S: 5′‐GCCGTTGGAGAAATCATGTCAC‐3′	147	57.0	38	Almhanna et al., (2022)
	AS: 5′‐TGGCAGCGTTCCAACAATTG‐3′				
MasR	S: 5′‐CTTTGTGGAGAACGGGAT‐3′	100	52.0	34	Costa‐Besada et al., (2018)
	AS: 5′‐GGAGATGTCAGCAATGGA‐3′				
eNOS	S: 5′‐CTGCGGTGATGTCACTATGG‐3′	140	57.2	34	Kwon et al., (2013)
	AS: 5′‐AAATGTCCTCGTGGTAGCGT‐3′				
GAPDH	S: 5′‐GTTACCAGGGCTGCCTTCTC‐3′	183	61	32	Wang et al., (2017)
	AS: 5′‐GATGGTGATGGGTTTCCCGT‐3′				

### 2.5 Statistical analysis

Data analysis used SPSS 26.0 software. One‐Way ANOVA and LSD post hoc test with 95% confidence interval (*p* < 0.05) were used to test the hypothesis.

## 3 RESULTS

### 3.1 ACE2 mRNA expressions in lung tissue of male Wistar rats

**FIGURE 1:**
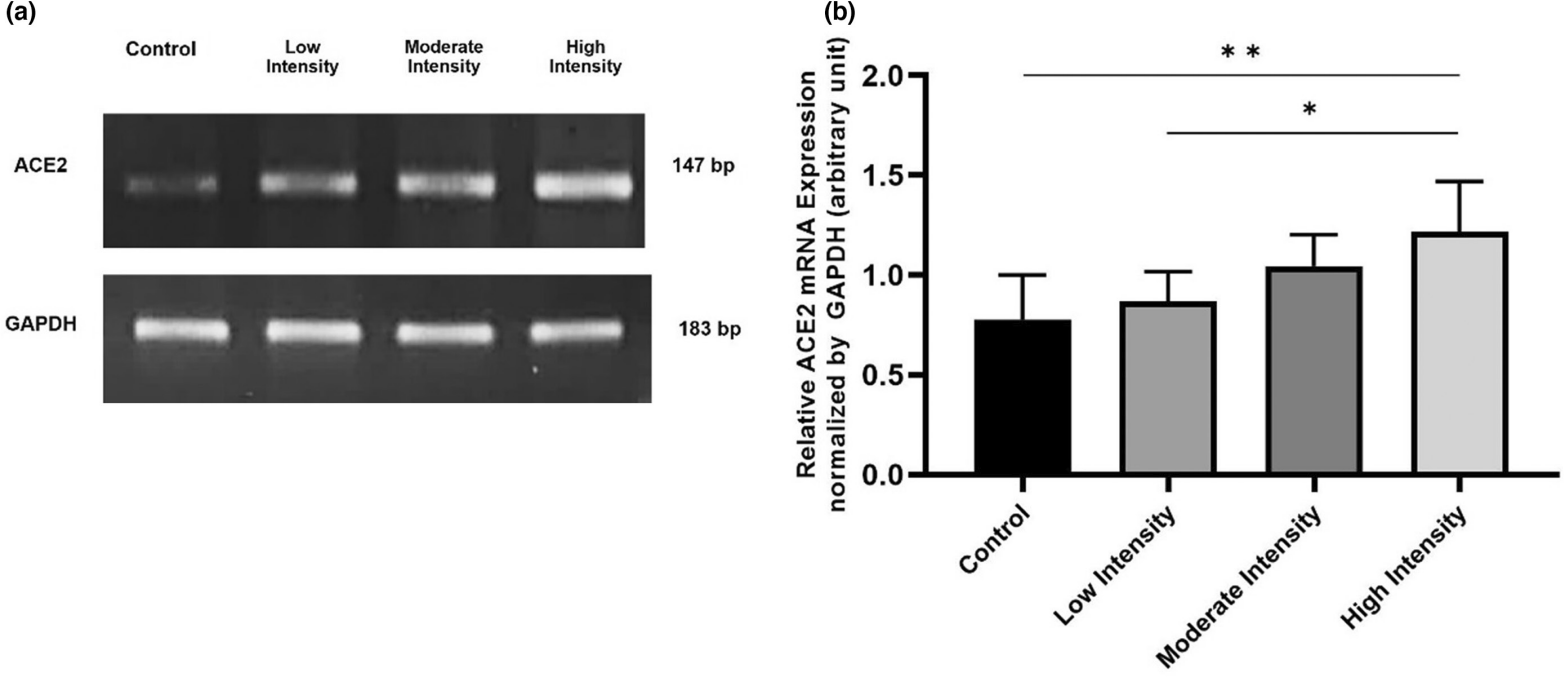
ACE2 mRNA expression in lung tissue after 8 weeks of aerobic exercise. (a) In rat lung tissue, different exercise intensities induce ACE2 mRNA expression. (b) High‐intensity exercise increased ACE2 mRNA compared to control and low‐intensity exercise. Data were analyzed using One‐Way ANOVA and LSD post hoc test. They expressed as mean ± standard error of the mean (SEM) with *p* < 0.05 considered as significant (*) and *p* < 0.01 considered as very significant (**).

To confirm the role of ACE2 in inducing NO synthesis, we used semiquantitative PCR to analyze the ACE2 mRNA expressions. Compared to the control (0.777 ± 0,112), high‐intensity training (1.216 ± 0.127) significantly increased ACE2 mRNA expression (*p* = 0.003). A significant increase was also found between low (0.869 ± 0.074) and high‐intensities exercises (*p* = 0.015) (Figure 1b). Compared to the control, moderate‐intensity training (1.044 ± 0.079) increased the expression of ACE2 mRNA but not significantly.

### 3.2 MasR mRNA expressions in lung tissue of male Wistar rats

**FIGURE 2:**
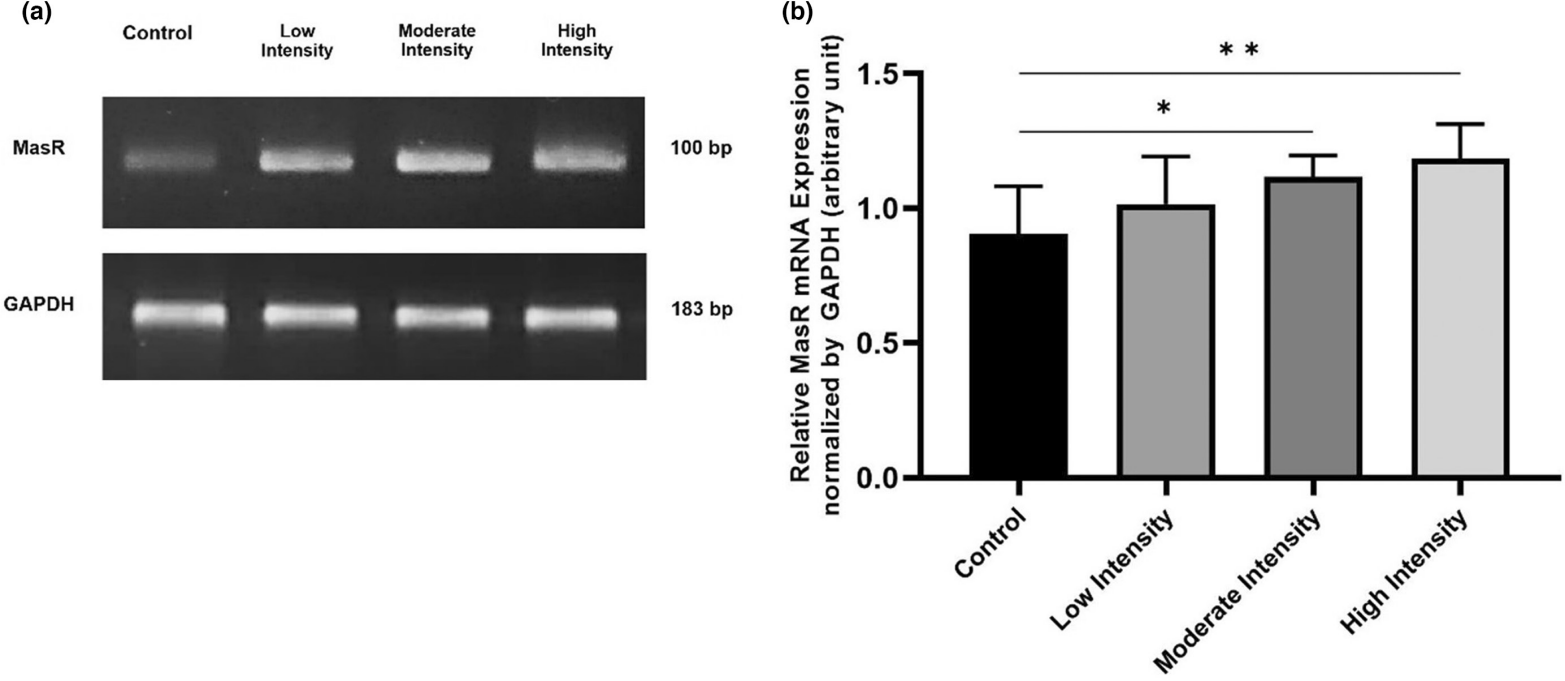
MasR mRNA expression in lung tissue after 8 weeks of aerobic exercise. (a) In rat lung tissue, different exercise intensities induce MasR mRNA expression. (b) Compared to control, high, and moderate‐intensity exercises increase MasR mRNA significantly. Data were analyzed using One‐Way ANOVA and LSD post hoc test. They expressed as mean ± standard error of the mean (SEM) with *p* < 0.05 considered as significant (*) and *p* < 0.01 considered as very significant (**).

We also analyzed MasR mRNA expression in the lung to identify the receptors involved in exercise‐induced NO synthesis. Compared to the control (0.904 ± 0.089), moderate (1.117 ± 0.040) and high‐intensity (1.186 ± 0.064) exercises caused a significant increase in MasR mRNA expressions (*p* = 0.035 and *p* = 0.008, respectively) (Figure 2b). Low‐intensity training (1.016 ± 0.089) increased the MasR mRNA expression but not significantly.

### 3.3 eNOS mRNA expressions in lung tissue of male Wistar rats

**FIGURE 3:**
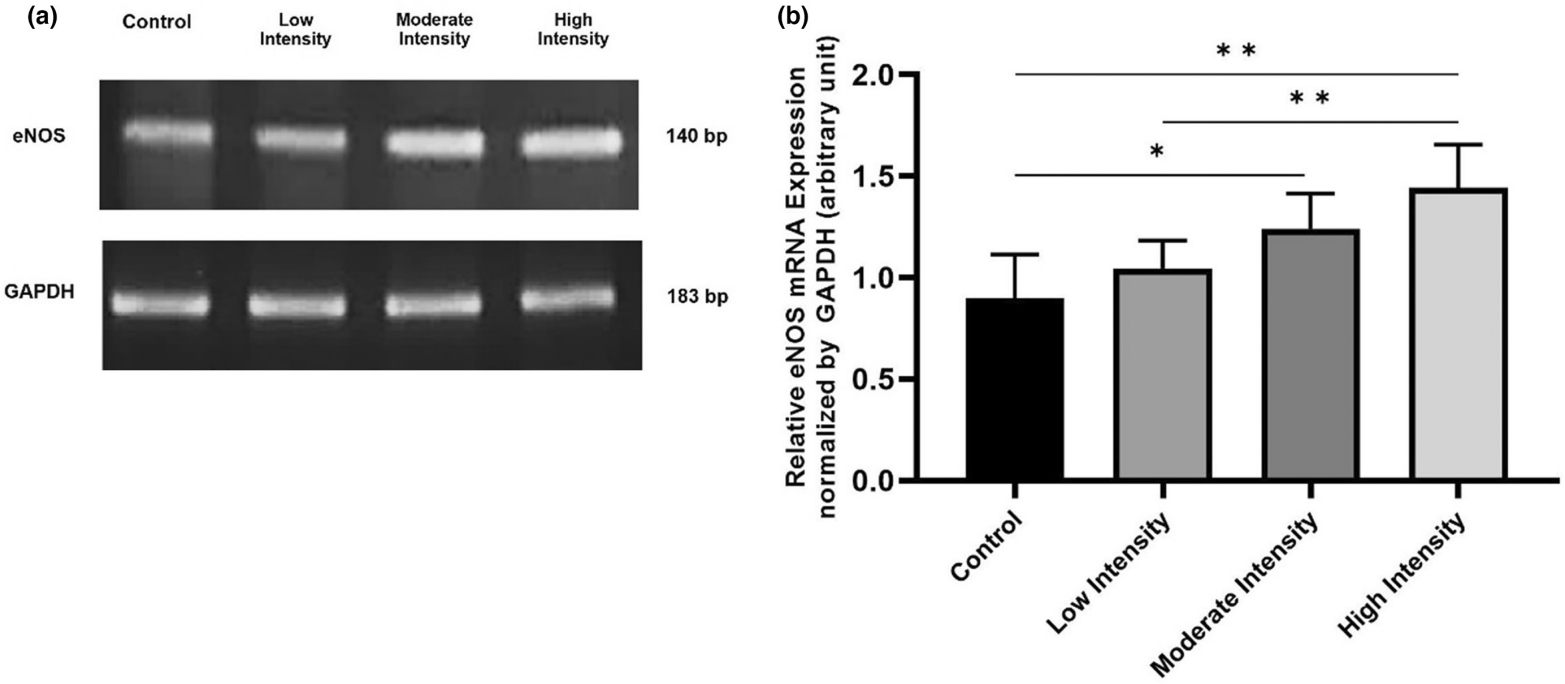
eNOS mRNA Expression in lung tissue after 8 weeks of aerobic exercise. (a) In rat lung tissue, different exercise intensities induce eNOS mRNA expression. (b) Compared to control, moderate, and high‐intensity exercises significantly increase eNOS mRNA expression. Data were analyzed using One‐Way ANOVA and LSD post hoc test. They expressed as mean ± standard error of the mean (SEM) with *p* < 0.05 considered as significant (*) and *p* < 0.01 considered as very significant (**).

As one of the enzymes most involved in synthesizing NO, eNOS mRNA expressions in lung tissue were also examined. Compared to the control (0.899 ± 0.107), moderate (1.241 ± 0.087) and high‐intensity (1.442 ± 0.107) exercise caused a significant increase in eNOS mRNA expression (*p* = 0.011 and *p =* 0.000). High‐intensity exercise also significantly increases eNOS mRNA expression compared to low‐intensity exercise (1.045 ± 0.069) (*p =* 0.004) (Figure 3b).

### 3.4 Effects of exercise intensity on muscular lung artery

**FIGURE 4:**
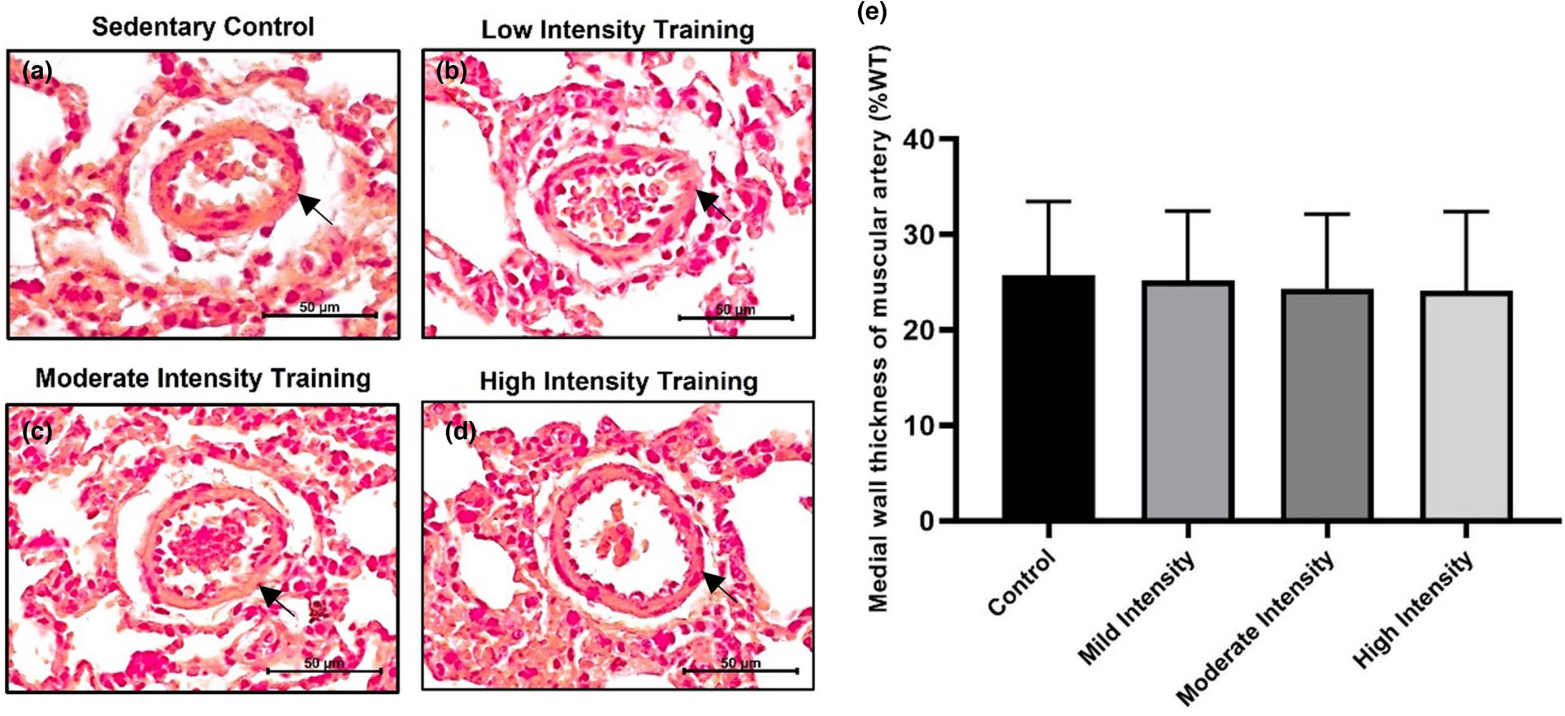
Effect of exercise intensity on medial wall thickness of muscular lung artery. (a–d) Representative microscopic figure of the muscular lung artery after hematoxylin and eosin staining. (e) Quantification of medial wall thickness of muscular artery (%WT). Data were analyzed using One‐Way ANOVA and LSD post hoc test. They expressed as mean ± standard error of the mean (SEM).

Exercise intensities created different effects on the medial wall thickness of the muscular artery (%WT). Low (25.220 ± 3.640), moderate (24.344 ± 3.881), and high‐intensity (24.130 ± 4.129) exercises decreased the medial wall thickness of lung arteries but not significantly. Compared to the control group (25.761 ± 3.844), high‐intensity exercise had the most optimal impact on reducing medial wall thickness (Figure 4).

## 4 DISCUSSION

The lung is a vital organ whose function is supported by the vascular component. One of the lung's closely related functions to the vascular role is perfusion. Vasodilation is one of the responses to regulate the level of lung perfusion. NO is one of the vasodilators that plays a role in this condition. Besides contributing to vasodilation, NO can also inhibit VSMC proliferation (Tanner et al., 2000). ACE2/MasR/eNOS pathway is one of the pathways that can induce NO synthesis. Different exercise intensities may create different effects on that pathway in lung tissue.

This study found that the ACE2/MasR/eNOS pathway may contribute to adaptive lung response toward high‐intensity exercise because a high‐intensity treadmill could increase the ACE2, MasR, and eNOS gene expression, contributing to NO synthesis. This study's findings align with previous research, which stated that high‐intensity aerobic exercise for 8 weeks increased the expression of ACE2 mRNA and NO_2_ in kidney tissue of spontaneously hypertensive rats (Almeida et al., 2020). Another study also showed that high‐intensity interval exercise caused a significant increase in plasma ACE2 levels (Magalhães et al., 2020). Previous studies also stated that exercise could increase the expression of the MasR and eNOS genes (Garcia et al., 2022; Gurovich et al., 2022; Silva et al., 2011). This condition possibly results from hypoxia following high‐intensity exercise, increasing ACE2 expression. A previous study stated that blood hypoxia induced the transmembrane ACE2(tACE2) expression in vascular endothelium during high‐intensity exercise (Abe et al., 2015; Hagiu, 2021). Furthermore, AMPK activity during high‐intensity exercise also influenced ACE2 phosphorylation. AMPK activity increased along with increased exercise intensity (Simpson, 1964). A previous study also stated that AMPK activation increased Mas receptor expression (Kim et al., 2018). In addition, endothelial shear stress (ESS) also induced eNOS expression. ESS is the vascular system's adaptation to blood flow changes during exercise. Previous studies have stated that moderate and high‐intensity exercise produces higher ESS than low‐intensity exercise (Padilla et al., 2008). The positive effects of high‐intensity exercise on vascular functions have been explained by some previous studies (Bond et al., 2015; Chidnok et al., 2020; Iwamoto, 2018).

Interestingly, in this present study, a moderate‐intensity treadmill only increases the MasR and eNOS mRNA expressions. This condition may occur because during moderate‐intensity exercise, there is no increase in ACE2 expression in vascular endothelial cells (Hagiu, 2021). In response to low‐intensity training, the expression of ACE2, MasR, and eNOS genes increased but not significantly. Low‐intensity exercise may require a longer time to increase the expression of these genes. A previous study explained that 10 weeks of low‐intensity training increased ACE2 and Ang‐(1–7) levels (Fernandes et al., 2011). The insignificant increase in ACE2 mRNA expression in the low and moderate groups is probably because low and moderate‐intensity exercise did not increase AMPK activity as high as the increase in AMPK occurring in high‐intensity exercise. Previous studies stated that this increase in AMPK occurred at exercise intensity >60% of maximal aerobic capacity (Simpson, 1964).

Our study shows that low‐, moderate‐, and high‐intensity treadmills decreased the muscular wall thickness of the muscular lung artery. These results align with previous studies, stating that high‐intensity training for 4 weeks decreased lung arterial wall thickness (Moreira‐Gonçalves et al., 2015). Another study showed that 5 weeks of treadmill running with low to moderate intensity could reduce the arterial wall thickness more in rats injected with monocrotaline (MCT) than those injected with MCT but did not get aerobic exercise (Colombo et al., 2013). Based on the data in this study, the effect of exercise intensity on lung arterial structure remodeling remains inconclusive. This condition can be because longer exercise duration must cause arterial remodeling. Previous studies explained that low‐moderate intensity exercise decreased wall‐to‐lumen ratio and brachial artery wall thickness significantly in humans who exercised for 24 weeks (Green et al., 2010). In addition, metabolic factors can also influence the results of this study. Previous studies stated that individuals with metabolic disorders had a higher intima‐media wall thickness (Cuspidi et al., 2018).

The limitation of this study is that we did not examine the protein expressions, lung functions, NO concentration, and the degree of vasodilation that might occur. This study has described the effect of exercise intensities on genes inducing NO synthesis in male Wistar rat lungs but may not apply in humans. We only used male Wistar rats as subjects, so we could not observe the effect of reproductive hormones on the results in this study. Future studies can be conducted to determine the optimal exercise intensity to increase lung fitness or VO_2_ max and vascular function.

In conclusions, differences in exercise intensity affect the ACE2/MasR/eNOS signaling pathway, which can induce NO synthesis in the lung. High‐intensity exercise may induce vasodilation by increasing mRNA expression of ACE2, MasR, and eNOS without decreasing the medial wall thickness of the muscular artery. In contrast, moderate‐intensity exercise may induce vasodilation by increasing mRNA expression of MasR and eNOS without reducing the medial wall thickness of the muscular artery. Thus, high‐intensity exercise may be the most optimal intensity to improve vascular function.

## AUTHOR CONTRIBUTIONS

Hanna Goenawan, Vita Murniati Tarawan, and Ronny Lesmana conceived and designed the study. Hanna Goenawan and Yani Medina Lestari acquired the data and drafted the manuscript. Hanna Goenawan, Yani Medina Lestari, and Ronny Lesmana, analyzed and interpreted the data. Achadiyani Achadiyani, Putri Teesa Radhiyanti, and Hamidie Ronald Daniel Ray revised the draft for intellectual content. All authors approved the final version of the manuscript.

## FUNDING INFORMATION

The study was supported by grant funding for H.G/V.M.T from Universitas Padjadjaran, grant number: 1549/UN6.3.1/PT.00/2023.

## CONFLICT OF INTEREST STATEMENT

The authors declare no competing of interest.

## ETHICS STATEMENT

This protocol was approved by the Research Ethics Committee of Padjadjaran University (1256/UN6.KEP/EC/2022).
